# Fins, fur, and wings: the study of Tmem161b across species, and what it tells us about its function in the heart

**DOI:** 10.1007/s00335-023-09994-z

**Published:** 2023-05-24

**Authors:** Kelly A. Smith, Nicole Dominado, Jessica F. Briffa

**Affiliations:** Department of Anatomy & Physiology, The University of Melbourne, Parkville, VIC 3010 Australia

## Abstract

Transmembrane protein 161b (Tmem161b) was recently identified in multiple high-through-put phenotypic screens, including in fly, zebrafish, and mouse. In zebrafish, Tmem161b was identified as an essential regulator of cardiac rhythm. In mouse, Tmem161b shows conserved function in regulating cardiac rhythm but has also been shown to impact cardiac morphology. Homozygous or heterozygous missense mutations have also recently been reported for TMEM161B in patients with structural brain malformations, although its significance in the human heart remains to be determined. Across the three model organisms studied to date (fly, fish, and mouse), Tmem161b loss of function is implicated in intracellular calcium ion handling, which may explain the diverse phenotypes observed. This review summarises the current knowledge of this conserved and functionally essential protein in the context of cardiac biology.

## Introduction

For vertebrates, the heart is responsible for sustaining life. Whilst across vertebrate species, the heart exhibits considerable anatomical diversity, there are also many universalities. These include the heart’s famous ability to beat with rhythmicity, and the interdependence of both form and function for correct development. Biomechanical forces generated by heart function (such as blood flow) have a direct impact on cardiac morphogenesis and, reciprocally, appropriate heart morphology is required for correct blood flow to occur (Andres-Delgado and Mercader 2016). Despite this, or possibly because of this co-dependency, there are molecular factors that are required for both processes in cardiac development: for correct cardiac morphogenesis and rhythm. In this brief report, we will explore one such factor, transmembrane protein 161b (Tmem161b).

Tmem161b is a recently identified protein containing eight transmembrane domains but no other recognisable domains. To date, its molecular function remains elusive. This is an exciting proposition: understanding a new regulator in cardiac development is likely to contribute new information about how heart development and rhythm is regulated at the molecular level. Evidence shows that Tmem161b is important for both heart morphology and rhythm.

### Identification of Tmem161b: a novel regulator of cardiac rhythm in zebrafish

From an ENU-based forward genetic screen in zebrafish, the *grime/tmem161b* (Gene ID ENSDARG00000055989) mutant was identified presenting with cardiac rhythm defects (Koopman et al. 2021). Homozygous mutant embryos showed reduced heart rate from the very onset of the heartbeat and, as embryos develop, showed increasing incidence of arrhythmic episodes. From 10 to 15 days post-fertilisation (dpf) homozygous mutant larvae die, although the cause of this lethality has not been established. Whilst a range of morphological features in the heart were examined in the *grime/tmem161b* mutant as well as a CRISPR-Cas9-generated allele, no differences in cardiac morphology were observed between homozygous mutant and wildtype embryos. This included quantification of cardiomyocyte number, cell shape and size, and differentiation of various structures within the heart (ventricle, atrium, atrioventricular canal, sinoatrial node) (Koopman et al. 2021). Together, these observations suggest that Tmem161b function in the zebrafish heart acts exclusively to regulate electrical activity, at least up until 5 dpf; the latest timepoint morphological analyses were performed.

Electrophysiological analyses of adult heterozygous *grime/tmem161b* hearts showed that Tmem161b has a considerable effect on cardiomyocyte activity: Tmem161b was required to inhibit I_Kr_ and I_CaL_ ion currents [potassium (K^+^) and calcium (Ca^2+^), respectively], both of which are essential in maintaining correct cardiac action potential dynamics and, therefore, cardiac rhythm. Importantly, analysis of Ca^2+^ transients using a gCaMP reporter line showed increased Ca^2+^ transient amplitude, suggesting that Ca^2+^ levels were increased in homozygous *grime/tmem161b* mutant cardiomyocytes (Koopman et al. 2021). Together, these data showed that Tmem161b is required to regulate cardiac ion currents at the plasma membrane and may also play a role in regulating intracellular Ca^2+^ levels or handling. However, the underlying mechanisms by which Tmem161b does so is yet to be determined.

## Functional roles of Tmem161b

### Tmem161b function in cardiac rhythm and ion regulation

**Fig. 1:**
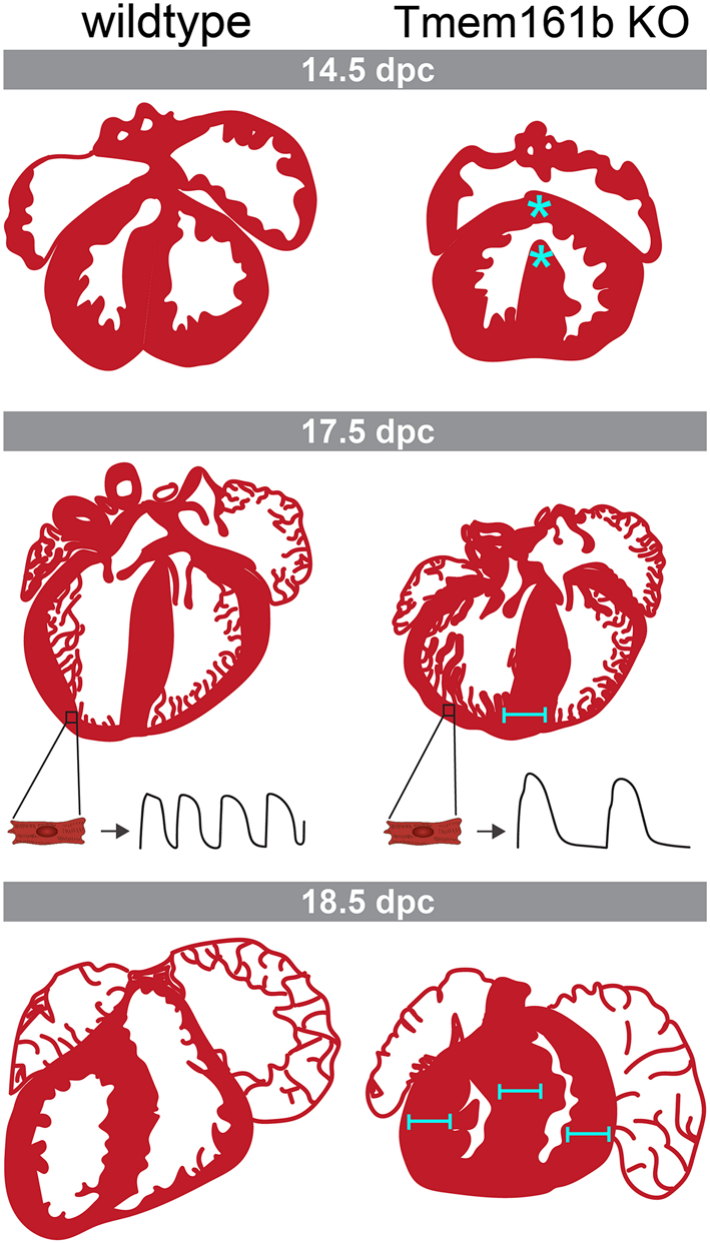
Summary of cardiac phenotypes observed in wildtype and TMEM161B knockout (KO) mouse embryos. At 14.5 dpc, TMEM161B KO embryos have VSDs and atrial and ventricular defects (AVSD, cyan asterisks). At 17.5 dpc, TMEM161B KO embryo hearts have altered morphology (although no reported VSD or AVSD), with thickened interventricular septum (cyan scale bar) and increased cell number. Isolated cardiomyocytes exhibit altered Ca.^2+^ oscillation, compared with wildtype littermates. At 18.5 dpc, the ventricular walls of TMEM161B KO embryonic hearts are considerable thicker (cyan scale bars) and, in some instances, almost entirely occlude the chamber lumen. Phenotypic summary derived from published data (Koopman et al. 2021; Spielmann et al. 2022) and IMPC data (http://www.mousephenotype.org/data/embryo)

Investigations into the conserved function(s) of Tmem161b was performed by the generation of global Tmem161b (Gene ID ENSMUSG00000035762) knockout mouse embryos. Ca^2+^ transients were examined in isolated cardiomyocytes by removing embryonic hearts [17.5 days post coitum (dpc)] and dissociating ventricles (Koopman et al. 2021). Cardiomyocytes from homozygous Tmem161b loss-of-function (LOF) mouse embryos had slower Ca^2+^ oscillations and increased variation between oscillations, consistent with phenotypes observed in *grime/tmem161b*-deficient zebrafish embryos. These changes in Ca^2+^ transients are consistent with increased intracellular Ca^2+^, although this was not examined (Fig. 1).

Interestingly, in a forward genetic mutagenesis screen in *Drosophila melanogaster* for tumour suppressors, the common ancestral Tmem161 gene, emei (Gene ID FBgn0036133), was also found to regulate Ca^2+^ levels (Ma et al. 2020). In their study, Ma et al. found that emei synergises with Ras oncogene at 85D (Gene ID FBgn0003205) (hereafter named RasV12) in both imaginal eye and wing discs, enhancing tumour growth and promoting invasion into neighbouring tissues. The downstream effectors of this growth are reported to be Hippo and JNK signalling. Increased cytosolic Ca^2+^ levels were required for homozygous mutant emei-RasV12 tumourigenesis, suggesting a common mechanism (Ma et al. 2020). Indeed, increasing cytosolic Ca^2+^ levels in combination with RasV12 [by disrupting SERCA (Gene ID FBgn0263006)] phenocopied emei-RasV12 mutants and, reciprocally, decreasing cytosolic Ca^2+^ levels [by Stromal interaction molecule (Stim) (Gene ID FBgn0045073) knockdown[ rescued the emei-RasV12 overgrowth phenotype. These data suggest that Tmem161 activity sits upstream of cytosolic Ca^2+^ levels and that Tmem161b plays a functional and conserved role in regulating Ca^2+^.

### Tmem161b function in cardiac morphology

In contrast to the electrical phenotype, a difference was observed between fish and mouse when examining cardiac morphology. Whilst no effect on cardiac morphology was observed between wildtype and *grime/tmem161b* homozygous mutant zebrafish hearts, a distinct morphological difference was observed in mice (Koopman et al. 2021; Spielmann et al. 2022). At 17.5 dpc, Tmem161b LOF mice had increased relative heart mass compared with wildtype littermates. Closer examination showed no difference in cell size and no evidence of fibrosis but an increase in cell number; suggesting Tmem161b LOF hearts undergo hyperplasia (Koopman et al. 2021) (Fig. 1). This was most obvious, morphologically, by increased thickness of the interventricular septum towards the apex of the heart (Koopman et al. 2021) (Fig. 1).

In an independent study that examined an impressive 3894 mouse mutant lines for cardiac morphogenesis defects, Tmem161b was also identified (Spielmann et al. 2022). This study used microCT data from the International Mouse Phenotyping Consortium (IMPC) to identify mouse models with severe congenital heart defects. Homozygous Tmem161b LOF embryos were analysed at 14.5 dpc and presented with ventricular septal defects (VSDs) as well as malformations affecting the vena cava, atria, ventricles, and mitral valve (Spielmann et al. 2022) (Fig. 1). This contrasts with the above report, which did not report VSD or similar malformations (Koopman et al. 2021; Spielmann et al. 2022). The discordance between the two reports may stem from the different developmental stages examined (14.5 versus 17.5 dpc). Indeed, microCT data of later stage embryos on the IMPC database support this notion: homozygous Tmem161b LOF hearts at 18.5 dpc have no VSDs but their chamber walls are markedly thicker, to the extent that the ventricular lumen is almost entirely occluded (http://www.mousephenotype.org/data/embryo) (Dickinson et al. 2016) (Fig. 1).

### Tmem161b function in other organ systems

In two recent back-to-back studies, the range of phenotypes in Tmem161b knockout mice was extended further (Akula et al. 2023; Wang et al. 2023). Tmem161b knockout mice showed holoprosencephaly (a failure of the forebrain hemispheres to separate) as well as a range of other midline fusion defects. These defects appeared fully penetrant but with variable expressivity, and included cleft lip/palate, asymmetric eye defects and even instances of cyclopia (Akula et al. 2023; Wang et al. 2023). Growth defects and perinatal lethality were also reported, corroborating an earlier report (Akula et al. 2023; Koopman et al. 2021; Wang et al. 2023). In developing brains, the cortical layer was thinner in knockout embryos and neurons were mispatterned, which were attributed to defects in either neural cell migration or cell fate acquisition (Akula et al. 2023; Wang et al. 2023). Analysis of spinal cords showed patterning defects in some dorsoventral domains, patterning known to be regulated by Sonic Hedgehog (Shh) signalling (Akula et al. 2023). This combination of phenotypes is highly suggestive of impaired Shh signalling or cilia defects and, indeed, severe structural defects were observed for cilia in Tmem161b knockout mouse brains (Akula et al. 2023). Despite this, several other stereotypical Shh or cilia phenotypes were not observed in these animals. For example, no limb or digit abnormalities were observed, nor skeletal dysplasia (Akula et al. 2023). This suggests either the central nervous system is particularly sensitive to TMEM161B-deficiency, or it is functioning differently in this tissue. Whichever the case, is currently unclear how TMEM161B is functioning molecularly to perturb Shh signalling or cilia structure.

## TMEM161B in human disease

TMEM161B was definitively shown to be required for human development in back-to-back publications. Akula et al. (2023) and Wang et al. (2023) demonstrated homozygous or compound heterozygous missense mutations in TMEM161B in multiple families with cortical folding malformations of the brain. Affected individuals presented at birth with polymicrogyria (excessive number of gyri or folds of the cerebral cortex), intractable seizures, microcephaly, and hypotonia (decreased muscle tone) (Akula et al. 2023; Wang et al. 2023). Individuals examined between 2 and 7 years of age showed developmental delay, motor skill deficits, speech impairment, and spastic quadriplegia (Akula et al. 2023; Wang et al. 2023). Disease modelling in mice via knock-in of a large proportion of patient alleles showed homozygosity for patient variants that were not as severe as knockout, suggesting that these mutations are hypomorphic (Wang et al. 2023). This marries well with population data from the gnomAD database, which identifies no individuals with homozygous LOF variants in TMEM161B, with the exception of a missense mutation in the stop codon, resulting in a slightly elongated protein by 57 amino acids (Chen et al. 2022). Whilst there is scant information about the cardiac phenotypes of the affected individuals described by Akula et al. and Wang et al., one individual died at age 32 from sudden cardiac death (Akula et al. 2023). Upon autopsy, this individual was reported with cardiomyopathy comprising mild hypertrophy and dilatation (Akula et al. 2023), phenotypes consistent with the mouse (Akula et al. 2023; Koopman et al. 2021). These data suggest that TMEM161B is essential for human survival and that, whilst individuals with hypomorphic homozygous mutations can survive, it is accompanied by severe neurological defects. The extent to which TMEM161B is required for cardiac development and physiology in humans remains unclear.

From population studies, TMEM161B function has also been associated with behavioural phenotypes. SNP risk variants near the TMEM161B locus have been identified for attention deficit/hyperactivity disorder (Liao et al. 2019) and genome-wide association studies (GWAS) have implicated it in major depressive disorder (Muench et al. 2018). Additionally, numerous epidemiological studies link deletions and rare duplications in a ~ 10 Mb region on chromosome 5q14.3 [where TMEM161B (Gene ID ENSG00000164180) is located] to neurological defects, developmental delay, hypotonia and seizures (Cardoso et al. 2009; Engels et al. 2009; Ilari et al. 2016; Le Meur et al. 2010; Novara et al. 2010; Nowakowska et al. 2010; Zweier et al. 2010). In many instances these phenotypes have been attributed to MEF2C-deficiency (Gene ID ENSG00000081189) (Ilari et al. 2016; Le Meur et al. 2010; Novara et al. 2010; Nowakowska et al. 2010; Zweier et al. 2010), which is adjacent to TMEM161B in the human genome. However, given the consistency of these phenotypes with those described by Akula et al. and Wang et al., disruption to TMEM161B may actually be the causative locus. Alternatively, it may be that deletion of both genes contributes to the observed phenotypes. What is clear is that TMEM161B is now a compelling candidate in these cases.

## TMEM161B-AS1

The TMEM161B locus lies on chromosome 5. This locus is conserved in the fly but not the fish or the mouse (https://lncipedia.org/db/gene/TMEM161B-AS1). Along the TMEM161B locus lies the divergent long non-coding RNA (lncRNA) TMEM161B-AS1. TMEM161B-AS1 has been reported to modulate proliferation, invasion, and migration in many cancers (Chen et al. 2021; Dong et al. 2019; Shi et al. 2021). Silencing TMEM161B-AS1 in human glioma cells inhibits proliferation, migration, and invasion. Conversely, proliferation, migration, and invasion are promoted in human oesophageal cancer upon downregulation of TMEM161B-AS1. In both studies, TMEM161B-AS1 is reported to function by acting as a sponge to microRNAs hsa-miR-27a-3p and miR-23a-3p, respectively. Whether TMEM161B-AS1 can modulate TMEM161B expression in a similar fashion has yet to be determined.

## Tmem161 protein family

Evolutionarily, a common ancestral Tmem161 gene is found in sponges (the most ancient of the animal kingdom), worm, fly, and jawless fishes. Tmem161 orthologues are also identified across Kingdoms, where they are found in at least Plantae. This suggests that the Tmem161 family is ancient and probably fulfils a fundamental cellular function. Duplication of Tmem161 occurred in jawed fishes, giving rise to Tmem161a and Tmem161b. This duplication coincides with segmentation of the heart and development of the pacemakers and conduction system. Both Tmem161b and Tmem161a are found in fishes, birds, and mammals.

As described above, Tmem161b has eight transmembrane domains with no other identifiable domains, and it is currently unknown what its molecular mechanism of action is. Unfortunately, further analysis into the Tmem161 protein family provides minimal additional information. Protein BLAST analysis suggests that Tmem161a is the only other Tmem161 protein family member and it is considerably divergent from Tmem161b: In humans, TMEM161A has 48% similarity to TMEM161B at the amino acid level (47% in mouse, 48% in zebrafish). Unfortunately, knowledge of Tmem161a function is equally mysterious. Like Tmem161b, Tmem161a has 8 predicted transmembrane domains with no other identifiable domains, and little is known about its function. Its expression has been reported to increase upon oxidative stress, and it is suggested to be protective against oxidative stress (Montesano Gesualdi et al. 2006); however, no mechanism for this has been described.

## Conclusions

This leaves us with the question: How can we reconcile these various phenotypes across different model systems and tissue contexts to provide a unifying picture of Tmem161b function? It is possible that Tmem161b performs more than one molecular function within cells. Alternatively, it may play a single functional role that impacts multiple-cellular processes that explains the seemingly distinct functions. Tmem161b has been implicated in growth and proliferation, regulation of action potentials, and in modulating Ca^2+^ transients and levels. In fact, Ca^2+^ handling is disrupted upon Tmem161 LOF in all contexts studied to date and, crucially, Ca^2+^ channelopathies can produce both cardiac and neurological disorders: such as in Timothy Syndrome (mutations in CACNA1C (Gene ID ENSG00000151067)) (Splawski et al. 2004). Whatever the function of Tmem161b, the cellular mechanism(s) likely impacts Ca^2+^ regulation in some way. Detailed biochemical analyses will no doubt assist in unravelling such details of the molecular mechanism of Tmem161b.
